# Glycoside Compounds from Blood-Nourishing Chinese Medicinal Herbs: Structural Characteristics, Pharmacological Mechanisms, and Therapeutic Potential for Thrombocytopenia

**DOI:** 10.3390/molecules31050894

**Published:** 2026-03-08

**Authors:** Jianqin Tang, Hai Li, Jun Du, Yanjun Zhang, Jianming Wu, Xi Du, Xiaoqin Zhang

**Affiliations:** 1 Key Laboratory of Medical Electrophysiology, Ministry of Education & Medical Electrophysiological Key Laboratory of Sichuan Province (Collaborative Innovation Center for Prevention of Cardiovascular Diseases), Institute of Cardiovascular Research, Southwest Medical University, Luzhou 646000, China; 20234099120032@stu.swmu.edu.cn (J.T.); 20244099120032@stu.swmu.edu.cn (H.L.); 2 School of Basic Medical Sciences, Southwest Medical University, Luzhou 646000, China; lzjundu@163.com (J.D.); zhangyanjun@swmu.edu.cn (Y.Z.)

**Keywords:** glycoside compounds, structural composition, thrombocytopenia, hematopoiesis, immunomodulation, medicinal plants

## Abstract

Thrombocytopenia is a common hematological disorder characterized by reduced platelet counts and an increased risk of bleeding, for which current pharmacological treatments are often limited by adverse effects, drug resistance, or high costs. Traditional Chinese medicinal herbs such as ginseng, notoginseng, peony root, and astragalus have long been used for blood-nourishing and qi-tonifying purposes and are frequently prescribed for conditions associated with blood deficiency and hematopoietic dysfunction. This review systematically summarizes glycoside compounds derived from these herbs, focusing on their structural characteristics and pharmacological activities relevant to thrombocytopenia. Accumulating evidence indicates that glycosylation enhances the solubility, bioavailability, and stability of aglycones, thereby influencing their biological effects. Preclinical studies suggest that glycoside compounds may improve the hematopoietic microenvironment through anti-inflammatory, antioxidant, and immunomodulatory actions, potentially reducing immune-mediated platelet destruction. In addition, they may promote thrombopoiesis by modulating hematopoietic signaling pathways, such as PI3K/AKT, and by restoring immune balance, particularly via regulation of the Treg/Th17 axis. Collectively, these multi-target effects on hematopoiesis and immune regulation highlight glycoside compounds as promising lead candidates for the development of novel therapeutic approaches to thrombocytopenia.

## 1. Introduction

Platelets play a central role in hemostasis and immune surveillance. Impaired platelet production or accelerated platelet destruction can lead to thrombocytopenia, commonly defined as a platelet count below 100 × 10^9^/L, which frequently occurs in patients receiving radiotherapy or chemotherapy, as well as in those with autoimmune disorders such as immune thrombocytopenia (ITP). Severe thrombocytopenia substantially increases the risk of spontaneous bleeding and may become life-threatening [1,2]. Current clinical treatments face several limitations, such as platelet transfusions carrying risks of alloimmunization and infection [3]. In contrast, glucocorticoids and thrombopoietin receptor agonists (such as eltrombopag) pose risks of thrombosis and are costly [4,5,6], and splenectomy involves significant surgical trauma [7,8]. Therefore, there is an urgent need to develop new, safe, and effective therapeutic approaches.

**Figure 1:**
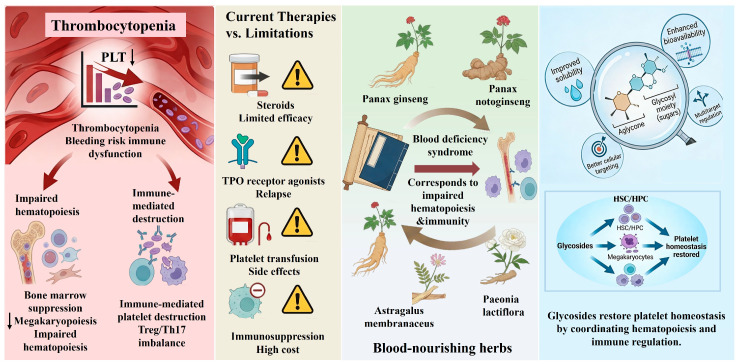
Schematic representation of thrombocytopenia pathogenesis, current therapeutic limitations, and glycoside-based intervention strategies.

In traditional Chinese medicine (TCM), thrombocytopenia-like conditions are commonly categorized under syndromes such as “blood deficiency” and “failure of blood to be retained,” which are characterized by fatigue, pallor, bleeding tendency, and delayed recovery after illness. Herbs such as Panax ginseng, Astragalus membranaceus, and Paeonia lactiflora have been traditionally prescribed to “nourish the blood” and control bleeding, indications that conceptually overlap with impaired hematopoiesis and immune dysregulation in modern medicine [9,10,11]. This correspondence provides an ethnopharmacological rationale for exploring these herbs as potential therapeutic resources for thrombocytopenia [12]. Experimental studies have suggested that such interventions may accelerate the recovery of white blood cells and platelets and ameliorate myelosuppression-related injury (Figure 1).

Modern phytochemical and pharmacological studies have demonstrated that glycosides constitute the major bioactive components of many blood-nourishing Chinese medicinal herbs. Representative examples include ginsenosides from Panax ginseng, astragalosides from Astragalus membranaceus, paeoniflorin from Paeonia lactiflora, and related saponins and flavonoid glycosides from other medicinal species. These compounds exhibit diverse biological activities, including anti-inflammatory, antioxidant, immunomodulatory, and hematopoietic effects, and have been shown to promote platelet production and attenuate immune-mediated platelet destruction in experimental models [13,14]. Importantly, the presence of glycosyl moieties markedly influences the solubility, stability, bioavailability, and cellular targeting of aglycones, thereby shaping their pharmacological profiles [15,16,17,18].

Accumulating evidence suggests that glycosides exert therapeutic effects on thrombocytopenia through coordinated regulation of hematopoietic stem and progenitor cells, megakaryocyte differentiation, immune cell function, and the bone marrow microenvironment. These effects have been consistently observed in models of bone marrow suppression, immune thrombocytopenia, and TCM-defined blood deficiency syndromes [19,20], providing mechanistic support for the traditional use of blood-nourishing herbs.

Despite increasing research interest in glycoside compounds derived from traditional Chinese medicinal herbs, existing studies remain fragmented and are often discussed separately from their chemical characteristics or ethnopharmacological background. A systematic integration linking glycoside structural features with their pharmacological actions in thrombocytopenia is still lacking. In this review, we focus on glycosides isolated from blood-nourishing Chinese medicinal herbs and summarize current evidence regarding their effects on platelet production, hematopoietic regulation, immune modulation, and the bone marrow microenvironment. The available experimental and clinical evidence is organized according to compound classes and biological processes, with particular attention to the role of glycosylation in shaping pharmacological properties. By bridging traditional medicinal concepts with modern pharmacological findings, this review aims to provide a coherent framework for understanding the therapeutic potential and limitations of glycoside-based interventions for thrombocytopenia treatment, and to support future research and rational drug development in this field.

## 2. Methodology of Literature Search

This narrative review was conducted based on a comprehensive literature search of peer-reviewed publications. Relevant studies were retrieved from PubMed, Web of Science, and Scopus databases using combinations of keywords including “thrombocytopenia”, “immune thrombocytopenia”, “glycosides”, “saponins”, “ginsenosides”, “astragalosides”, “blood-nourishing”, and “traditional Chinese medicine”.

The search covered publications from January 2012 to December 2025. Original research articles, review articles, and clinical studies reporting the chemical structures, pharmacological activities, mechanisms of action, or safety profiles of glycoside compounds related to thrombocytopenia were included. Studies unrelated to hematopoiesis, platelet regulation, or immune-mediated platelet disorders were excluded.

The selected literature was further categorized according to glycoside classes, structural features, hematopoietic mechanisms, immunomodulatory effects, and translational relevance, which formed the basis for the thematic organization of this review.

## 3. Application of Chinese Herbal Medicine in Thrombocytopenia

### 3.1. Corresponding Traditional Chinese Medicine Syndromes for Thrombocytopenia

Within the traditional Chinese medicine (TCM) framework, conditions resembling thrombocytopenia are not defined as a single disease entity but are generally classified under syndromes related to blood deficiency and disordered blood containment. Clinically, Blood Deficiency Syndrome is characterized by fatigue, pale complexion, dizziness, spontaneous bleeding, purpura, and delayed recovery following illness or chemotherapy. These manifestations closely resemble platelet deficiency and impaired hematopoietic function as described in modern medicine. However, in traditional Chinese medicine clinical practice, thrombocytopenia-related manifestations are not exclusively attributed to Blood Deficiency. Pathogenic patterns such as Blood Heat, characterized by inflammation-driven bleeding and accelerated platelet consumption, are also frequently recognized, particularly in immune-mediated thrombocytopenia.

According to TCM theory, the pathogenesis of Blood Deficiency Syndrome is primarily associated with dysfunction of the spleen and kidney systems, insufficiency of qi and blood, and impaired regulation of defensive qi. These factors collectively contribute to bleeding tendencies, reduced hematopoietic capacity, and weakened recovery following injury or treatment. Accordingly, therapeutic principles emphasize “nourishing the blood,” “tonifying qi to generate blood,” and “regulating immune balance to prevent bleeding,” forming the theoretical basis for the long-standing application of blood-nourishing herbal medicines in hematological disorders [21].

Although derived from traditional diagnostic concepts, these principles show notable conceptual convergence with contemporary understandings of thrombocytopenia, including impaired hematopoiesis, immune-mediated platelet destruction, and dysfunction of the bone marrow microenvironment.

### 3.2. Herbal Medicines for Blood Deficiency Syndromes in Traditional Chinese Medicine

A wide range of traditional Chinese medicinal herbs has been extensively documented in classical medical literature and modern clinical practice for the management of blood deficiency-related syndromes [22,23]. Representative examples include *Panax ginseng*, *Panax notoginseng*, *Paeonia lactiflora* (white and red peony root), *Astragalus membranaceus*, *Rehmannia glutinosa* (raw and prepared forms), and *Sanguisorba officinalis*. Rather than functioning through direct supplementation of blood components, these herbs operate within a holistic regulatory framework that emphasizes the harmonization of qi and blood, the balance of yin and yang, and the restoration of systemic homeostasis.

From a functional perspective, these herbs may be broadly categorized according to their predominant roles in supporting qi, regulating circulation, and limiting pathological bleeding, although such classifications remain conceptual rather than absolute and are applied flexibly in clinical practice.

From a traditional perspective, ginseng occupies a central position in the concept that “qi generates blood,” exerting its effects primarily by tonifying primordial qi and strengthening spleen and stomach function rather than directly replenishing blood itself [11,22]. This traditional concept is supported by modern pharmacological evidence showing that ginsenoside Rg1 regulates hematopoietic stem/progenitor cell proliferation and differentiation [24]. In clinical practice, ginseng-containing formulations such as Dushen Decoction and Renshen Yangrong Tang have been widely used for chronic anemia, post-chemotherapy bone marrow suppression, and postoperative recovery, reflecting their role in promoting endogenous hematopoietic recovery.

Notoginseng demonstrates distinctive therapeutic characteristics described as “arresting bleeding without causing stasis, and resolving stasis without damaging vital essence,” addressing the pathological coexistence of blood deficiency and blood stasis [25,26,27]. By improving microcirculation and alleviating stasis, notoginseng creates favorable conditions for effective hematopoiesis while avoiding excessive stagnation associated with simple blood-tonifying approaches.

*Sanguisorba officinalis* is traditionally applied to “cool the blood and arrest hemorrhage,” particularly in conditions involving concurrent blood deficiency and bleeding [28,29]. By limiting ongoing blood loss and protecting mucosal and hematopoietic tissues, it indirectly supports hematopoietic recovery in bleeding-prone states such as chemotherapy-associated thrombocytopenia [29,30].

The medicinal use of peony exemplifies the principle of “same origin, different effects.” White peony root nourishes blood, preserves yin, and alleviates liver hyperactivity, whereas red peony root clears heat, cools the blood, and resolves stasis, making each suitable for distinct pathological patterns [31,32,33]. Astragalus, a classical representative of “tonifying qi to generate blood,” has been widely used in formulas such as Danggui Buxue Decoction to treat chemotherapy-induced myelosuppression and various forms of anemia, with increasing pharmacological evidence supporting its hematopoietic and immunoregulatory effects [23,34,35,36].

Rehmannia further illustrates the TCM concept of preparation-dependent functional divergence: raw rehmannia clears heat and cools the blood, whereas prepared rehmannia primarily nourishes blood, replenishes essence, and supports marrow function, forming a core component of classical blood-nourishing formulas such as Si-Wu-Tang [34,37,38].

Collectively, these herbs constitute a therapeutic system that extends beyond simple blood supplementation, emphasizing coordinated regulation of hematopoietic function, immune balance, microcirculation, and the hematopoietic microenvironment. Through precise herb selection and synergistic formulation, TCM provides a comprehensive framework for managing diverse blood-related syndromes, including blood deficiency, blood stasis, blood heat, and bleeding-prone conditions [11,22,39].

### 3.3. From Traditional Herbal Practice to Chemical Material Basis

Although the therapeutic effects of blood-nourishing Chinese medicinal herbs have been validated through long-standing clinical application, these effects are increasingly attributed to specific classes of bioactive constituents identified through modern phytochemical studies. Among these, glycoside compounds have emerged as major contributors to the hematopoietic and immunomodulatory activities of blood-nourishing herbs.

Rather than acting through isolated molecular targets, glycosides exhibit integrated regulatory effects that are consistent with the holistic treatment principles of traditional Chinese medicine. Their widespread distribution across blood-nourishing herbs, combined with their structural diversity and favorable physicochemical properties, positions glycosides as a critical molecular bridge between traditional therapeutic concepts and modern pharmacological mechanisms.

Accordingly, a systematic examination of the structural features and physicochemical properties of glycoside compounds is essential for understanding how traditional herbal interventions translate into measurable biological effects. This consideration provides the rationale for the following section, which focuses on the structure and properties of glycosides and establishes the chemical foundation for subsequent discussions of their pharmacological mechanisms in thrombocytopenia.

## 4. Structure and Properties of Glycosides

Glycosides represent the principal bioactive constituents of many traditional Chinese medicinal herbs prescribed for blood-nourishing purposes, including Panax ginseng, Astragalus membranaceus, Paeonia lactiflora, and related species. From a pharmacological perspective, the defining feature of glycosides is the covalent linkage between a sugar moiety (glycone) and a non-sugar moiety (aglycone), a structural arrangement that critically determines their physicochemical properties, biological targeting, and therapeutic activity in hematopoietic and immune systems.

### 4.1. Sources and Pharmacological Activities of Glycosides

Glycosides are widely distributed in nature, and many bioactive constituents of medicinal plants exist predominantly in glycosylated forms. These compounds can be extracted from herbal materials using solvent reflux extraction, ultrasound-assisted extraction, microwave-assisted extraction, or enzymatic hydrolysis. Glycosides exhibit diverse pharmacological activities, including immunomodulatory, anti-tumor, antioxidant, hypoglycemic, and cardiovascular protective effects, as summarized in Table 1.

Importantly, despite sharing common pharmacological profiles, glycoside compounds display substantial heterogeneity in their biological activities, which cannot be fully explained by their botanical origin alone. Increasing evidence indicates that variations in glycosylation degree, sugar composition, glycosidic linkage type, and attachment position critically influence their bioactivity, target selectivity, and pharmacokinetic behavior.

**Figure 2:**
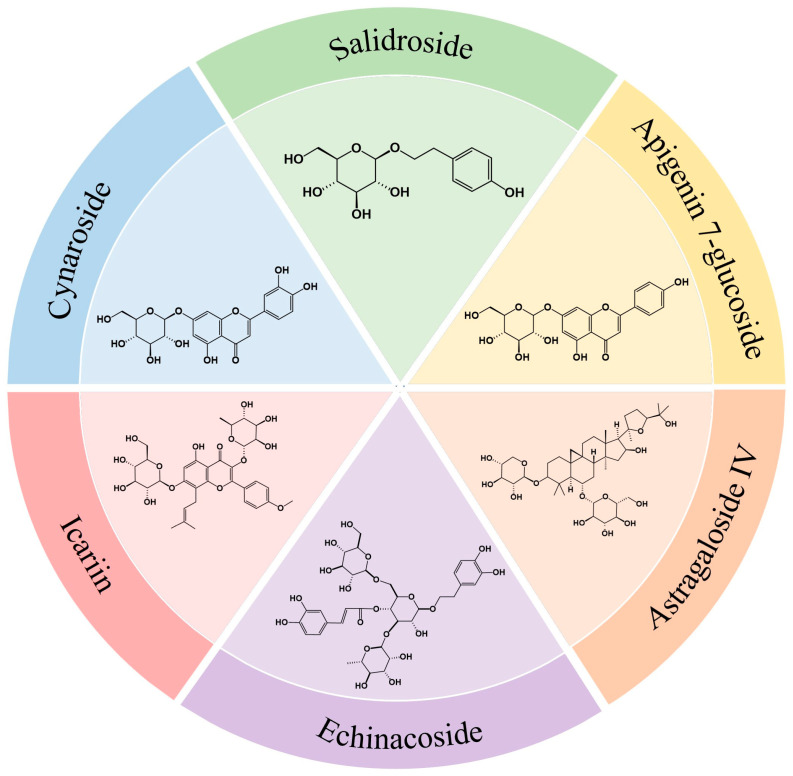
The chemical structures of glycoside compounds such as salidroside, apigenin 7-glucoside, astragaloside, echinacoside, icariin, and baicalin.

Structurally, glycosides derived from blood-nourishing Chinese medicinal herbs can be broadly categorized based on their aglycone scaffolds and glycosylation patterns, including triterpenoid saponins (e.g., ginsenosides and astragalosides), monoterpene glycosides (e.g., paeoniflorin), and flavonoid glycosides. Representative structural frameworks and glycosylation features of these glycoside classes are illustrated in Figure 2.

Given this pronounced structural diversity, a structure–activity relationship (SAR)-oriented analysis is essential to elucidate how specific glycosylation features contribute to thrombopoietic efficacy and immunomodulatory effects. Accordingly, the following section focuses on the SAR characteristics of glycoside compounds in the context of thrombopoiesis.

### 4.2. Structure–Activity Relationships of Glycoside Compounds in Thrombopoiesis

Accumulating evidence indicates that the biological activities of glycoside compounds in thrombocytopenia are strongly influenced by their structural characteristics, particularly the number, type, and linkage position of sugar moieties, as well as the nature of the aglycone backbone. These structure–activity relationships (SARs) play critical roles in modulating hematopoietic signaling, immune regulation, and pharmacokinetic behavior.

#### 4.2.1. Influence of Glycosylation Degree and Sugar Composition

The degree of glycosylation represents a key determinant of glycoside bioactivity. In general, mono- or di-glycosylated compounds tend to exhibit higher biological potency than highly glycosylated analogues, likely due to improved membrane permeability and receptor accessibility [60]. For example, among ginsenosides, less-polar protopanaxadiol-type saponins with fewer sugar residues have been reported to display stronger regulatory effects on hematopoietic cell proliferation and differentiation, whereas highly glycosylated derivatives show reduced activity, possibly owing to steric hindrance and limited cellular uptake [28,42].

The composition of sugar moieties further modulates glycoside activity. Glucose-containing glycosides are the most prevalent and often exhibit favorable bioactivity profiles, whereas rhamnose- or arabinose-containing glycosides may alter molecular stability and metabolic behavior, thereby influencing biological outcomes [60]. Such structural variations contribute to differential regulation of platelet production and immune responses, as observed in astragalosides and paeoniflorin derivatives [31,33,53].

#### 4.2.2. Effect of Glycosylation Position on Biological Activity

The position at which sugar moieties are attached to the aglycone scaffold critically affects biological function. In ginsenosides, glycosylation at the C-3 and/or C-20 positions has been shown to significantly alter hematopoietic activity. Compounds glycosylated at C-3 often exhibit enhanced effects on megakaryocyte differentiation, whereas additional sugar substitutions at C-20 may attenuate receptor interaction and downstream signaling activation [28,61]. These findings suggest that precise control of glycosylation sites is essential for optimizing thrombopoietic activity.

Similarly, in paeoniflorin-related glycosides, the glycosidic linkage at specific hydroxyl positions contributes to their immunomodulatory properties, influencing inflammatory cytokine production and oxidative stress responses that are closely associated with platelet survival and destruction [31,32,46].

#### 4.2.3. O-Glycosides Versus C-Glycosides

The nature of the glycosidic bond also affects stability and activity. O-glycosides are more susceptible to enzymatic hydrolysis in vivo, which may facilitate the release of active aglycones but also limit systemic exposure. In contrast, C-glycosides exhibit enhanced metabolic stability and prolonged circulation time, potentially leading to sustained biological effects. Studies on flavonoid and saponin C-glycosides suggest that increased stability may contribute to improved immunoregulatory and anti-inflammatory activities relevant to thrombocytopenia [62].

However, the enhanced stability of C-glycosides may also reduce metabolic flexibility, indicating that an optimal balance between stability and bioactivation is necessary for therapeutic efficacy.

#### 4.2.4. Impact of Glycosylation on Pharmacokinetics and Translational Potential

Beyond intrinsic bioactivity, glycosylation markedly influences pharmacokinetic properties. Sugar moieties generally enhance aqueous solubility and chemical stability, facilitating formulation and systemic administration. Nonetheless, many glycoside compounds, particularly saponins, still suffer from poor oral bioavailability due to limited intestinal absorption and extensive first-pass metabolism [63].

It has been hypothesized that glycosylation may enable interactions with glucose transporters or lectin-like receptors, thereby enhancing tissue distribution [64]; it should be emphasized that these transporter-related mechanisms remain largely hypothetical, as direct experimental evidence supporting selective accumulation of glycosides in the bone marrow hematopoietic niche is currently limited [65]. Therefore, such mechanisms should currently be regarded as speculative rather than fully validated.

Collectively, these SAR analyses highlight that rational modulation of sugar number, composition, linkage type, and attachment position is crucial for optimizing the thrombopoietic and immunomodulatory activities of glycoside compounds. Notably, comparative structure–activity evidence remains fragmentary, and in many cases is derived from indirect comparisons rather than systematic sugar-removal or site-specific modification studies, highlighting a key limitation of current SAR interpretations. A deeper understanding of these relationships will facilitate the design of glycoside-based lead compounds with improved efficacy and translational potential for thrombocytopenia therapy.

### 4.3. Regulation of Hematopoietic Signaling Pathways by Glycoside Compounds

Multiple studies indicate that glycoside compounds promote thrombopoiesis primarily through the modulation of hematopoietic signaling pathways rather than direct platelet replacement. In cellular and animal models, these compounds have been shown to activate key pathways involved in megakaryocyte proliferation and differentiation, including PI3K/AKT [66,67], MAPK/ERK [68], and JAK/STAT [69] signaling cascades [61,70,71].

Activation of the PI3K/AKT pathway plays a central role in regulating megakaryocyte survival, polyploidization, and cytoskeletal remodeling, processes that are essential for effective platelet production [72,73]. Several glycoside compounds derived from ginseng and astragalus have been reported to enhance AKT phosphorylation, thereby promoting megakaryocyte maturation and increasing platelet output in thrombocytopenia models [74,75,76].

In parallel, modulation of the JAK/STAT pathway contributes to hematopoietic homeostasis by regulating cytokine responsiveness and megakaryocyte lineage commitment [77]. Glycoside-mediated attenuation of excessive STAT3 activation has been associated with reduced inflammatory stress within the bone marrow microenvironment, indirectly supporting platelet production [69,71].

Collectively, these findings suggest that glycoside compounds exert their thrombopoietic effects through coordinated regulation of multiple hematopoietic signaling pathways, thereby restoring platelet production capacity under pathological conditions.

### 4.4. Immunomodulatory Effects of Glycoside Compounds: Linking Immune-Mediated Thrombocytopenia and “Blood-Heat” Theory

Beyond their effects on hematopoietic signaling, glycoside compounds exert pronounced immunomodulatory activities that are highly relevant to immune-mediated thrombocytopenia, particularly immune thrombocytopenic purpura (ITP). From an integrative perspective, immune-mediated thrombocytopenia, such as immune thrombocytopenic purpura (ITP), can be partially interpreted as a modern biomedical correlate of the traditional “Blood-Heat” pattern, in which excessive inflammation, immune activation, and oxidative stress drive platelet destruction. This conceptual alignment provides a theoretical framework for linking TCM pattern differentiation with contemporary immunopathological mechanisms. In modern biomedical terms, excessive inflammatory activation and immune imbalance represent key drivers of platelet destruction, which conceptually correspond to the traditional Chinese medicine notion of “Blood-Heat” [78,79,80].

Experimental evidence demonstrates that glycoside compounds suppress pro-inflammatory cytokine production, including IL-6, IL-17, and TNF-α, while inhibiting NF-κB and ROS-related signaling pathways [81,82,83]. These effects contribute to the attenuation of inflammation-driven platelet clearance and oxidative damage within the hematopoietic microenvironment.

Importantly, several glycoside compounds have been reported to restore immune homeostasis by regulating the balance between regulatory T cells (Treg) and Th17 cells [84,85,86], a critical axis in the pathogenesis of ITP [87]. By enhancing Treg differentiation and suppressing Th17-mediated inflammation, these compounds reduce immune-mediated platelet destruction and support platelet survival.

From an integrative perspective, the anti-inflammatory and immune-balancing effects of glycoside compounds provide a mechanistic bridge between the traditional concept of “Blood-Heat” and contemporary immunopathological models of thrombocytopenia, highlighting their dual therapeutic potential in both platelet protection and production.

### 4.5. Safety Profiles and Translational Considerations

Safety evaluation represents a critical prerequisite for the clinical translation of glycoside compounds. Existing evidence from in vitro and in vivo studies suggests that most glycoside compounds derived from blood-nourishing medicinal herbs exhibit low cytotoxicity and favorable tolerability profiles at therapeutically relevant doses [88,89,90]. In summary, the properties, linkage types, and quantity of sugar moieties collectively constitute the “structure–activity code” of glycoside compounds. This serves as both the fundamental basis for their diverse pharmacological activities and represents the scientific core behind the precise efficacy of traditional medicinal herbs.

Animal studies have generally reported minimal hepatotoxicity, nephrotoxicity, or hematological adverse effects following administration of representative glycosides, supporting their relative safety compared with conventional thrombopoietic agents [91,92,93]. Moreover, preliminary clinical observations indicate acceptable safety margins in formulations containing glycoside-rich extracts, although rigorous controlled trials remain limited [94,95,96].

Despite these encouraging findings, several translational challenges remain. Poor oral bioavailability, metabolic instability, and potential batch-to-batch variability of herbal-derived glycosides may limit clinical applicability [97,98]. Additionally, the risk of pro-thrombotic effects associated with excessive platelet stimulation warrants careful dose optimization and long-term safety assessment.

Therefore, future studies should focus on pharmacokinetic optimization, standardized formulation strategies, and well-designed clinical trials to fully evaluate the therapeutic potential and safety of glycoside compounds in thrombocytopenia management.

### 4.6. Integrated Structure–Mechanism Basis of Glycoside-Mediated Thrombopoietic Effects

Collectively, the pharmacological effects of glycoside compounds in thrombocytopenia are determined by an integrated interplay between their structural characteristics and multi-level biological mechanisms. As discussed above, variations in glycosylation degree, sugar composition, linkage type, and attachment position critically shape the bioactivity and target engagement of glycosides, thereby influencing their downstream hematopoietic and immunomodulatory effects.

From a mechanistic perspective, glycosides do not act through a single pathway but rather exert coordinated regulation across multiple biological processes, including promotion of megakaryocyte proliferation and differentiation, attenuation of immune-mediated platelet destruction, and modulation of inflammatory and oxidative stress signaling. Notably, distinct structural classes of glycosides tend to exhibit preferential mechanistic profiles. For example, triterpenoid saponins are more closely associated with thrombopoietic signaling and hematopoietic support, whereas monoterpene and flavonoid glycosides frequently display pronounced immunoregulatory and anti-inflammatory activities.

This structure–mechanism coupling provides a plausible molecular basis for the multitarget and synergistic therapeutic effects of blood-nourishing traditional Chinese medicinal herbs in thrombocytopenia. Importantly, it also highlights that the observed pharmacological outcomes cannot be attributed solely to herbal origin or individual signaling pathways, but rather arise from the collective action of structurally diverse glycoside constituents.

On this basis, the following section will further examine how these glycoside-mediated mechanisms are manifested across different pathological contexts of thrombocytopenia, with particular emphasis on immune dysregulation, inflammatory signaling networks, and disease-specific models.

## 5. Pharmacological Mechanisms of Glycoside Compounds in the Treatment of Thrombocytopenia

Glycoside compounds derived from traditional Chinese medicinal herbs have been shown, predominantly in preclinical models, to modulate thrombocytopenia-related pathological processes through coordinated regulation of hematopoiesis, immune homeostasis, and the bone marrow microenvironment. Rather than acting on a single molecular target, these compounds influence multiple interconnected biological pathways that collectively govern platelet production, survival, and functional recovery under pathological conditions. It should be noted that the majority of mechanistic insights summarized in this section are derived from cellular and animal models, whereas direct evidence from well-controlled human studies remains relatively limited.

**Figure 3:**
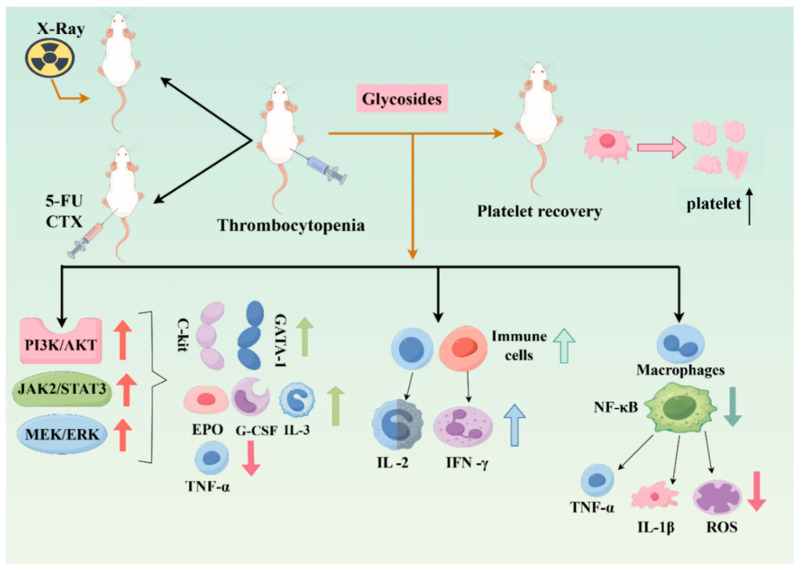
Mechanism of glycoside compounds in treating thrombocytopenia.

As illustrated in Figure 3, in mouse models of thrombocytopenia induced by radiotherapy or chemotherapy (e.g., cyclophosphamide or 5-fluorouracil), glycoside compounds have been reported to activate key hematopoietic signaling pathways, including MEK/ERK, JAK2/STAT3, and PI3K/AKT [66,68,99]. These signaling events are accompanied by the upregulation of transcription factors such as *c-Kit* and *GATA-1*, modulation of cytokine networks, increased serum levels of erythropoietin (EPO), granulocyte colony-stimulating factor (G-CSF), and interleukin-3 (IL-3), as well as suppression of tumor necrosis factor-α (TNF-α) [61,91,100]. Together, these effects contribute to enhanced proliferation of hematopoietic stem cells and maturation of megakaryocytes, consistent with, and providing a modern pharmacological interpretation of, the traditional concept of “blood nourishment.”

In addition to hematopoietic regulation, glycoside compounds have been reported to promote the proliferation of splenic immune cells, macrophages, and T lymphocytes [46,101,102]. By modulating nonspecific immune responses, they may further enhance helper T-cell activation and increase the expression and secretion of interleukin-2 (IL-2) and interferon-γ (IFN-γ) [103,104]. Concurrently, inhibition of NF-κB signaling reduces the release of pro-inflammatory cytokines such as TNF-α and IL-1β, indirectly attenuating reactive oxygen species (ROS) generation and oxidative stress-induced platelet damage [105,106,107,108]. Collectively, these mechanisms provide a pharmacological basis for the traditional medicinal principle of “nourishing blood and regulating immunity.”

### 5.1. Promotion of Hematopoietic Cell Proliferation and Differentiation

**Figure 4:**
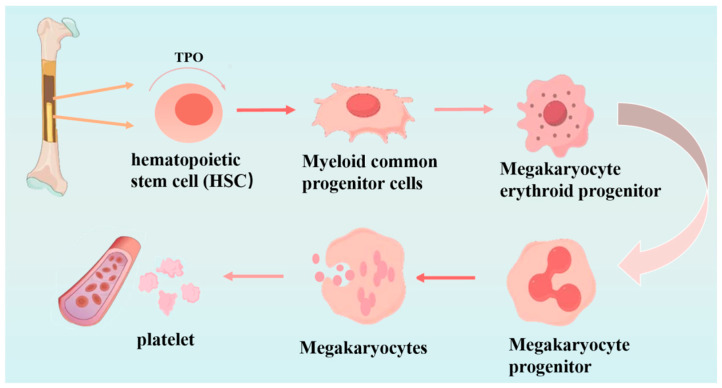
Schematic diagram of the platelet generation process.

Under physiological stimulation by thrombopoietin (*TPO*), hematopoietic stem cells (HSCs) in the bone marrow differentiate into megakaryocyte progenitor cells, which subsequently mature into megakaryocytes. During this maturation process, megakaryocytes undergo cellular enlargement, polyploidization, and cytoplasmic remodeling, ultimately releasing platelets into the circulation through cytoplasmic fragmentation, as schematically illustrated in Figure 4 [109,110].

#### 5.1.1. Promotion of Megakaryocyte Proliferation and Differentiation

Platelets originate from megakaryocytes derived from hematopoietic stem cells through a tightly regulated differentiation cascade. Accumulating evidence suggests that specific glycoside compounds can stimulate megakaryocyte proliferation and maturation, thereby enhancing platelet production.

For example, kaempferol-3-*O*-α-L-(4″-*E*-*p*-coumaroyl) rhamnoside (KCR) has been shown to upregulate GATA-1 expression via activation of the PKCδ/ERK1/2 signaling pathway, inducing megakaryocytic differentiation of progenitor cell lines such as K562 and HEL [13]. Similarly, cyanidin-3-*O*-β-glucoside promotes megakaryocyte generation, maturation, and proplatelet formation in vitro [70]. The glycoside compound 3,3′-dimethyl ellagic acid-4′-*O*-glucoside (DMAG), isolated from *Sanguisorba officinalis*, enhances the expression of platelet-associated markers including *ITGA2B*, *ITGB3*, *VWF*, and *PLEK*, while activating PI3K/Akt signaling to promote megakaryocyte differentiation and platelet production [66,68].

Collectively, these studies indicate that glycoside compounds facilitate megakaryocyte maturation through multiple intracellular signaling pathways; however, these observations are predominantly derived from in vitro systems and animal models.

#### 5.1.2. Promotion of Hematopoietic Stem/Progenitor Cell Proliferation and Differentiation

The proliferation and lineage commitment of hematopoietic stem/progenitor cells (HSPCs) play a critical role in sustaining platelet production. HSCs reside within a specialized bone marrow niche that tightly regulates their self-renewal and differentiation potential [111]. Modulation of HSPC proliferation and differentiation can increase megakaryocyte numbers and maturity, thereby enhancing platelet output [77,112,113].

Several glycoside compounds have been reported to promote hematopoiesis and thrombopoiesis by regulating HSPC function [114,115]. Panax notoginseng saponins (PNS) significantly stimulate the proliferation of CD34^+^ hematopoietic stem cells and promote directional differentiation toward granulocytic and erythroid lineages [116]. In addition, PNS exhibit growth factor–like activity by enhancing the effects of hematopoietic cytokines such as EPO, granulocyte–macrophage colony-stimulating factor (GM-CSF), and stem cell factor (SCF) [61,117]. Ginsenoside Rg1 has similarly been shown to regulate HSPC proliferation and differentiation through modulation of inflammatory mediators, including TNF-α and IL-6, and signaling pathways such as NF-κB and PI3K/Akt/mTOR [24,118].

#### 5.1.3. Promotion of Bone Marrow Cell Proliferation and Recovery from Myelosuppression

Myelosuppression, a common adverse effect of chemotherapy and radiotherapy, is characterized by impaired proliferation and differentiation of bone marrow hematopoietic cells, leading to reductions in red blood cells, white blood cells, and platelets [119,120]. Several glycoside compounds have demonstrated protective or restorative effects against myelosuppression in preclinical models.

Ginsenosides alleviate chemotherapy- or radiation-induced myelosuppression by promoting hematopoietic cell proliferation, regulating immune function, and exerting anti-inflammatory and antioxidant effects [120]. Ginsenosides Re and Rk3 significantly increase hemoglobin levels, bone marrow mononuclear cell counts, and platelet numbers in cyclophosphamide-treated mice, while modulating cytokine balance and inhibiting apoptosis-related pathways [91]. Rhodiola glycosides similarly enhance the proliferation of bone marrow progenitor cells by upregulating hematopoietic growth factors such as G-CSF and EPO, thereby accelerating hematopoietic recovery [19,121].

#### 5.1.4. Improvement of the Hematopoietic Microenvironment

The hematopoietic microenvironment comprises diverse cellular components, including endothelial cells, mesenchymal stromal cells, macrophages, and osteoblasts, as well as extracellular matrix molecules and cytokines [122,123,124]. Alterations in this microenvironment can profoundly influence megakaryocyte development and platelet production [125].

Glycoside compounds improve the hematopoietic microenvironment by modulating cytokine profiles, reducing oxidative stress, and supporting stromal cell function. Panax notoginseng total saponins stimulate stromal and immune cells to secrete erythroid and hematopoietic regulatory factors, thereby promoting progenitor cell proliferation [26]. Hydroxysafflor yellow A restores hematopoietic stem/progenitor cell proliferation and differentiation by suppressing excessive p53 activation and scavenging ROS, ultimately improving hematopoietic function in models of zinc deficiency or chemotherapy-induced injury [126].

Taken together, glycoside compounds promote hematopoietic cell proliferation, differentiation, and platelet generation through coordinated regulation of intracellular signaling pathways and the bone marrow microenvironment, as summarized in Table 2.

### 5.2. Immune Regulation

Immune thrombocytopenia (ITP) is an autoimmune disorder characterized by immune-mediated platelet destruction and impaired platelet production [133,134]. Dysregulated immune responses—including Th1/Th2 imbalance, defective regulatory T-cell function, abnormal cytotoxic T-cell activation, and pathogenic autoantibody production—are central to disease pathogenesis [135,136].

Notably, many inflammatory and oxidative pathways implicated in immune-mediated thrombocytopenia—such as IL-6/STAT3, NF-κB, IL-17, and reactive oxygen species (ROS) signaling—conceptually overlap, to some extent, with the traditional Chinese medicine description of “Blood-Heat” patterns. Increasing evidence suggests that Blood-Heat syndrome can be interpreted, from a modern biomedical perspective, as a pathological state characterized by excessive inflammation, immune activation, and redox imbalance, particularly involving dysregulated cytokine networks and T-cell-mediated immune responses [78,79,80].

**Figure 5:**
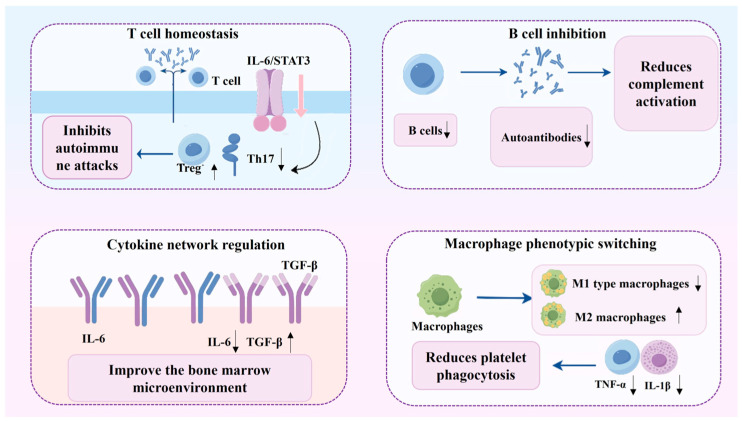
Immune regulatory mechanism of glycoside compounds in the treatment of thrombocytopenia.

As schematically summarized in Figure 5, glycoside compounds regulate immune homeostasis through multiple mechanisms, including modulation of T-cell differentiation, inhibition of aberrant B-cell activation, regulation of macrophage polarization, and attenuation of inflammatory and oxidative stress responses.

Accordingly, the immunomodulatory effects of glycoside compounds in thrombocytopenia can be broadly discussed in terms of direct regulation of immune cell function and attenuation of immune-related oxidative stress, as outlined below.

#### 5.2.1. Regulation of Immune Cell Function

In preclinical models, glycoside compounds have been shown to mitigate immune-mediated platelet destruction by modulating both innate and adaptive immune responses, including T-cell and B-cell activity, macrophage function, and cytokine signaling networks. Through these coordinated immunoregulatory effects, glycosides help restore immune homeostasis in immune-related thrombocytopenic conditions.

Icariin, a flavonoid glycoside derived from *Epimedium* species, exerts systemic immunomodulatory effects by regulating cytokine networks and suppressing inflammatory and oxidative stress–related signaling pathways, thereby reducing immune-mediated tissue injury [137,138,139]. In immune thrombocytopenia (ITP) models, ginsenoside PDS-C has been reported to inhibit macrophage-mediated phagocytosis of antibody-coated platelets while simultaneously promoting megakaryocyte proliferation and differentiation, thereby improving platelet recovery [102,140].

Tripterygium wilfordii polyglycosides, a class of immunosuppressive glycosides extracted from *Tripterygium wilfordii*, display potent anti-inflammatory and immunomodulatory properties. Both experimental and clinical studies have demonstrated their efficacy in ITP, which is attributed to the regulation of T-cell subsets, suppression of aberrant immune activation, and attenuation of inflammatory responses [141,142,143].

Monoterpene glycosides from *Paeonia lactiflora*, particularly paeoniflorin and albiflorin, have been shown to counteract blood deficiency states induced by chemotherapeutic agents through immune modulation. These compounds promote the secretion of hematopoietic growth factors, including G-CSF, GM-CSF, and PDGF-α, by bone marrow stromal cells while inhibiting hematopoietic suppressive factors, thereby supporting hematopoietic recovery and platelet production [10,144,145]. Similarly, rehmannioside D, a major glycoside component of prepared *Rehmannia glutinosa*, restores immune homeostasis by regulating immune cell function and cytokine secretion and has been reported to significantly increase platelet counts and bone marrow cellularity in murine models of blood deficiency [146].

In addition, glycoside-mediated immunoregulation may contribute to hematopoietic improvement in immune-related bone marrow failure conditions such as aplastic anemia. Ginsenoside Rg1, for instance, has been shown to protect hematopoietic stem cells by inhibiting mitochondria-mediated apoptosis, providing mechanistic insight into its potential role in immune-associated cytopenias [147,148,149].

#### 5.2.2. Attenuation of Immune-Related Oxidative Stress

Accumulating evidence indicates that oxidative stress contributes to immune-mediated thrombocytopenia by exacerbating platelet dysfunction, immune activation, and inflammatory signaling cascades [150,151,152]. Clinical observations have reported reduced antioxidant capacity and elevated markers of lipid peroxidation in patients with immune thrombocytopenia (ITP), suggesting an imbalance between pro-oxidant and antioxidant systems [153,154]. Importantly, oxidative stress is increasingly recognized as a pathophysiological modifier rather than a sole causative factor, acting in concert with immune dysregulation to aggravate platelet destruction and impaired thrombopoiesis [155,156].

Glycoside compounds possess intrinsic antioxidant and anti-inflammatory properties that may help attenuate oxidative stress–associated immune injury [157,158]. Through modulation of redox-sensitive signaling pathways, these compounds can indirectly protect platelets and hematopoietic cells from oxidative damage while dampening excessive immune activation [159,160,161,162]. For example, astragaloside IV has been shown to exert synergistic antioxidant and anti-inflammatory effects by regulating HIF-1α/NF-κB signaling, thereby reducing the production of pro-inflammatory cytokines and reactive oxygen species in preclinical models [163,164]. Such effects may contribute to the restoration of immune homeostasis and improvement of platelet survival under inflammatory conditions.

From a traditional medicine perspective, oxidative stress-related inflammatory responses conceptually overlap with the “Blood-Heat” pattern described in Chinese medicine, which is characterized by excessive inflammation and immune disturbance [80,165]. While this correspondence provides a useful interpretive framework, it should be emphasized that current evidence linking oxidative stress modulation by glycosides to clinical outcomes in thrombocytopenia remains largely preclinical and associative [160,166].

Overall, attenuation of immune-related oxidative stress represents a supportive and complementary mechanism by which glycoside compounds may contribute to the management of immune-mediated thrombocytopenia. When integrated with their regulatory effects on hematopoietic signaling and immune cell function, these antioxidant actions form part of a multi-target pharmacological network underlying the platelet-protective effects of glycosides, as summarized in Table 3. Nevertheless, further studies integrating redox biology, immune profiling, and clinical evaluation are required to clarify the translational relevance of these antioxidant effects [60].

## 6. Safety of Glycoside Compounds in the Treatment of Thrombocytopenia

Safety evaluation is a critical prerequisite for the translational development of glycoside-based interventions for thrombocytopenia. As core bioactive constituents of traditional medicinal herbs such as Panax ginseng and Astragalus membranaceus, glycoside compounds are characterized by multi-target and multi-pathway regulatory profiles. This pharmacological pattern is consistent with the traditional medicinal concept of holistic regulation and is often associated with relatively low toxicity and favorable tolerability.

Compared with limitations of currently used clinical therapies—such as infection risk associated with long-term glucocorticoid use or thrombotic complications linked to thrombopoietin receptor agonists—glycoside compounds have demonstrated a comparatively favorable safety profile in preclinical and early clinical studies. Importantly, however, this perceived safety advantage is derived primarily from traditional usage experience and emerging pharmacological evidence, and therefore requires systematic validation under modern regulatory and clinical frameworks.

### 6.1. Safety Evidence from Pharmacological Studies

In vitro studies indicate that glycoside compounds generally exhibit low cytotoxicity toward normal hematopoietic cells within pharmacologically effective concentration ranges. For example, ginsenoside Rg1 promotes hematopoietic stem cell proliferation and differentiation at concentrations of 10–100 μmol/L without inducing apoptosis or impairing cell viability [24]. Similarly, pinecone chrysanthemum glycosides stimulate bone marrow stromal cell secretion of GM-CSF without affecting cell cycle progression or viability [144,172]. Epimedium glycosides have also been reported to preferentially modulate abnormally activated signaling pathways, such as Hippo and NF-κB, while exerting minimal interference with physiological signaling in normal bone marrow cells [173,174,175].

Animal studies further support the relatively low toxicity of glycoside compounds derived from traditional medicinal herbs. In cyclophosphamide-induced myelosuppressed mouse models, ginsenosides Re and Rk3 significantly restored platelet counts and hematopoietic function without inducing detectable hepatic or renal toxicity, as assessed by serum ALT and BUN levels [91,100]. Rehmannioside D similarly improved hematopoiesis in blood-deficient mouse models without causing adverse outcomes such as weight loss or organ atrophy [176]. Notably, even under relatively high-dose administration—for example, panaxadiol saponin fractions at 50 mg/kg/day in immune thrombocytopenia models—no overt thrombotic events or excessive immunosuppression were observed [102,168]. Together, these findings suggest a relatively wide therapeutic window for glycoside compounds in preclinical settings.

### 6.2. Safety Validation in Clinical Studies

Accumulating clinical evidence suggests that glycoside-based formulations exhibit acceptable safety and tolerability in the treatment of thrombocytopenia, with generally mild and manageable adverse reactions. Several preparations have progressed into early clinical application, demonstrating safety profiles that are, in some aspects, more favorable than those of conventional Western pharmacotherapies.

#### 6.2.1. Clinical Safety of Single-Ingredient Formulations

Shengxueling Tablets, which contain total ginsenosides as the principal active components, have demonstrated significant platelet count improvement in patients with immune thrombocytopenia (ITP). Clinical observations reported no notable gastrointestinal intolerance, hepatic or renal dysfunction, or excessive immunosuppression during treatment, indicating good patient compliance and tolerability [177].

Similarly, ginsenoside diol (PDS-C), the active constituent of the traditional Chinese medicine formulation Painengda Capsule, exhibited favorable safety profiles in Phase I and II clinical studies. Oral administration at doses of 20–40 mg/day was effective in patients with ITP and chronic neutropenia, without increasing thrombotic risk or exacerbating bleeding symptoms. Compared with thrombopoietin receptor agonists such as eltrombopag, PDS-C may be more suitable for long-term maintenance therapy, although direct comparative trials remain limited [168,178].

#### 6.2.2. Safety of Combination Therapy

Guided by the traditional principle of toxicity-reducing compatibility, combination therapy involving glycoside compounds and conventional Western drugs has shown potential to enhance efficacy while mitigating adverse effects. For instance, combining Tripterygium wilfordii glycosides with recombinant human interleukin-11 (rhIL-11) in ITP treatment shortened the onset of platelet recovery by 3–5 days compared with rhIL-11 monotherapy, while reducing treatment-related adverse reactions such as fever and fatigue [142,143,179].

In newly diagnosed ITP patients, the combination of Painengda Capsules with corticosteroids allowed for a 30–50% reduction in corticosteroid dosage, accompanied by a lower incidence of steroid-associated adverse effects, including hyperglycemia and osteoporosis [168]. These findings provide clinical support for the concept of efficacy enhancement and toxicity reduction through rational combination therapy.

### 6.3. Current Limitations and Directions for Optimization in Safety Research

Despite encouraging safety signals, several limitations remain in the current body of evidence. First, most clinical studies have focused on short-term treatment durations (typically 3–6 months), and data on long-term safety—particularly regarding sustained immune modulation and bone marrow microenvironment homeostasis—are limited. Second, safety variability among glycoside compounds from different sources, such as herbal extraction versus chemical synthesis, has not been systematically characterized, highlighting the need for standardized quality control and pharmacokinetic evaluation. Third, although traditional medicine emphasizes syndrome differentiation, differences in safety and tolerability of glycoside compounds among patient subtypes (e.g., blood-deficiency versus blood-heat patterns) have not been adequately investigated. Taken together, these limitations underscore the need for future studies integrating long-term clinical evaluation, pharmacokinetic optimization, immune profiling, and TCM pattern stratification. Such efforts will be essential to rigorously define the safety boundaries and translational feasibility of glycoside-based interventions for thrombocytopenia.

Future safety optimization may benefit from integrating traditional pharmaceutical processing concepts with modern drug development strategies. For example, traditional processing methods aimed at toxicity reduction—such as the transformation of glycoside profiles during the preparation of Rehmanniae Radix Praeparata—may inform rational structural modification and glycosyl optimization approaches [17,57]. In addition, the development of multi-component formulations based on toxicity-reducing compatibility principles may allow a dose reduction of individual components while maintaining or enhancing therapeutic efficacy. Collectively, these strategies provide a conceptual and practical framework for improving the biosafety of glycoside compounds and facilitating their transition from traditional constituents to modern therapeutic agents.

## 7. Limitations & Translational Challenges

Despite the encouraging pharmacological profiles of glycoside compounds derived from blood-nourishing Chinese medicinal herbs, several important limitations and translational challenges should be carefully considered when interpreting the current evidence and evaluating their therapeutic potential for thrombocytopenia.

### 7.1. Limitations of Current Preclinical Evidence

At present, the majority of evidence supporting the thrombopoietic and immunomodulatory effects of glycoside compounds is derived from cell-based assays and animal models. Although these studies provide valuable mechanistic insights into megakaryocyte differentiation, platelet production, and immune regulation, they may not fully recapitulate the complex pathophysiology of human thrombocytopenia, particularly immune thrombocytopenia (ITP) [180]. Differences in disease etiology, immune system complexity, and drug metabolism between experimental models and human patients may limit the direct extrapolation of preclinical findings to clinical settings.

In addition, many animal studies employ acute or chemically induced thrombocytopenia models, whereas clinical thrombocytopenia often presents as a chronic and heterogeneous condition influenced by multiple immunological and inflammatory factors. Therefore, the therapeutic efficacy observed in preclinical models should be interpreted with caution [181].

### 7.2. Incomplete Structure–Activity Understanding and Study Heterogeneity

Although emerging structure–activity relationship (SAR) analyses suggest that glycosylation degree, sugar composition, linkage type, and attachment position critically influence bioactivity, the current SAR understanding remains fragmentary and context-dependent. Many studies investigate individual compounds under different experimental conditions, making direct comparisons across glycoside classes challenging.

Moreover, certain mechanistic interpretations—such as enhanced cellular uptake or tissue distribution mediated by glycosylation—are often inferred rather than experimentally validated [182]. For example, hypotheses proposing transporter-mediated recognition of O-glycosides should currently be regarded as speculative in the absence of direct in vivo evidence [183]. Future studies employing systematic SAR-guided design, standardized biological assays, and quantitative pharmacological evaluation are required to establish robust causal relationships between glycosylation patterns and thrombopoietic efficacy.

### 7.3. Pharmacokinetic and Safety Challenges

From a translational perspective, pharmacokinetic limitations represent a major barrier to the clinical development of glycoside compounds. Many saponin-type glycosides exhibit poor oral bioavailability, limited intestinal absorption, and extensive first-pass metabolism [184]. These characteristics may necessitate high dosing or alternative delivery strategies, which could increase the risk of adverse effects.

Although available preclinical and limited clinical data suggest an overall favorable safety profile, potential risks—including hepatotoxicity, immune overstimulation, and pro-thrombotic effects—cannot be fully excluded, particularly during long-term administration [185]. Comprehensive toxicological evaluations and pharmacokinetic–pharmacodynamic (PK–PD) studies are therefore essential before advancing glycoside compounds toward clinical application.

### 7.4. Gaps in Clinical Evidence and Translational Development

Clinical evidence supporting the use of isolated glycoside compounds for thrombocytopenia remains limited. Most human studies involve multi-herbal formulations or traditional prescriptions, making it difficult to attribute therapeutic effects to specific glycoside constituents. Furthermore, standardized clinical trials assessing dosage, efficacy, safety, and comparative effectiveness relative to existing therapies (e.g., TPO receptor agonists) are still scarce [180].

To facilitate clinical translation, future research should prioritize well-designed clinical studies, standardized compound preparation, and integration of modern pharmacological evaluation with traditional therapeutic concepts. Addressing these challenges will be critical for bridging the gap between preclinical promise and clinical utility.

## 8. Conclusions and Future Perspectives

This review systematically summarizes glycoside compounds derived from blood-nourishing traditional Chinese medicinal herbs, highlighting their structural characteristics, pharmacological mechanisms, safety profiles, and therapeutic potential in thrombocytopenia. By integrating traditional theoretical concepts with contemporary hematological and immunological evidence, glycosides emerge as promising multi-target candidates capable of modulating both platelet production and immune-mediated platelet destruction.

Nevertheless, as discussed in the preceding sections, the translation of glycoside compounds into clinically applicable therapies remains constrained by several challenges, including incomplete mechanistic validation, suboptimal pharmacokinetic performance, and the scarcity of well-designed clinical trials. Addressing these limitations will require interdisciplinary efforts that integrate medicinal chemistry, systems pharmacology, pharmacokinetics, and clinical research.

Future investigations focusing on rational structural optimization, improved drug delivery strategies, and high-quality clinical studies will be critical for advancing glycoside-based therapeutics from traditional medicine-derived lead compounds toward evidence-based interventions for thrombocytopenia.

## Figures and Tables

**Table 1:** Sources and pharmacological effects of glycoside compounds.

Medicinal Herbs	Glycosides	Extraction Method	Pharmacological Actions	Reference
*Panax ginseng*	Ginsenoside	Soxhlet extraction, hot reflux extraction.	Immune modulation, anti-tumor, antioxidant, and antibacterial.	[40]
*Panax notoginseng*	Panax notoginsenoside	Ethanol reflux method, enzymatic hydrolysis method, supercritical fluid extraction method.	Anti-cancer, anti-inflammatory, anti-tumor, antioxidant, anti-thrombotic, and treatment of cardiovascular diseases.	[28,41,42]
*Epimedium*	Icariin	ethanol extraction, ultrasonic extraction.	Boost immune function, protect the cardiovascular system, and provide antioxidant benefits.	[43,44,45]
*Paeonia*	Paeoniflorin, Paeoniflorin lactone	solvent heating, reflux, ultrasonic extraction, and microwave solvent extraction.	Protects nerves, heart, and brain blood vessels, lowers blood sugar and regulates immunity.	[33,46,47]
*Rhodiola rosea*	Salidroside	Water reflux extraction method, ethanol reflux extraction method, eutectic solvent method, dual-phase water extraction.	Anti-inflammatory, antioxidant, anti-cancer, immune-modulating, and cardiovascular disease treatment.	[48,49,50]
*Cistanches*	Echinacoside	Immersion extraction, reflux extraction, Soxhlet extraction, and ultrasonic extraction.	Antioxidant, antitumor, and immunomodulatory.	[51,52]
*Astragalus*	Astragaloside	Reflux extraction method, ultrasound-assisted extraction.	Anti-inflammatory, antioxidant, and cardioprotective.	[53,54,55]
*Rehmannia glutinosa*	Rehmannioside D	Methanol heating reflux extraction method.	Bone marrow hematopoiesis, enhanced immune function, and anti-inflammatory.	[56,57,58,59]

**Table 2:** Mechanism of glycoside compounds promoting hematopoietic cell proliferation and differentiation.

Glycosides	Cell Type	Result & Mechanism	Reference
Ginsenosides	Hematopoietic stem cells (HSCs), hematopoietic progenitor cells (HPCs).	Regulate HSC proliferation, differentiation, and migration through multiple targets (ACE, CaSR) and multiple pathways (Wnt/β-catenin, p53-p21, SDF-1/CXCR4).	[24]
Panax notoginseng total saponins	CD34^+^ cells and bone marrow nucleated cells (HL-60, K562, CHRF-288, and Meg-01 cells).	Induces the synthesis of GATA transcription factors, enhances their DNA-binding activity, upregulates the expression of genes associated with hematopoietic cell proliferation, and promotes hematopoiesis and blood cell formation.	[61,117]
Ginsenosides	Hematopoietic cells in bone marrow/CD34^+^ hematopoietic stem/progenitor cells	Synergistic hematopoietic growth factors promoted the expansion of CD34^+^ HSC/HPC in vitro. Promote the differentiation of CD34^+^ HSC/HPC into erythroid cells and promote the differentiation of CD34^+^ HSC/HPC into granulocytes.	[127]
Icariin	bone marrow cell	By acting on UCHL5, TNFAIP8, and Visfatin targets, activates IRS1/Akt/mTOR/GSK3β and cell cycle pathways and inhibits Hippo, JAK/STAT, and NF-κB pathways.	[128]
Apigenin 7-glucoside and Luteolin 7-glucoside	CD34^+^ hematopoietic stem/progenitor cells	Activation of the JAK/STAT signaling pathway induces erythroid differentiation of CD34^+^ cells and inhibits myeloid differentiation.	[129]
Kaempferol-3-*O*-α-‘L-(4′-*E*-*p*-coumaroyl) rhamnoside	HEL and K562 cells	Activation of the PKCδ/ERK1/2 signaling pathway induces erythroleukemia cells to differentiate into megakaryocytes and inhibits leukemogenesis.	[13]
cyanidin-3-*O*-β-glucoside	Meg-01 megakaryocyte cell	Cy-3-g promotes activated platelet apoptosis and enhances megakaryocyte proliferation, differentiation, and pro-platelet formation in vitro.	[70]
Panax diol saponins	Bone marrow megakaryocyte progenitor cells, megakaryocyte CHRF-288, and Meg-01 cell lines.	Through multi-target transcriptional regulation (GATA-1/RUNX1/NDRG2) and differentiation marker activation (CD41/CD42b), synergistically promote megakaryocyte progenitor cell proliferation and terminal differentiation.	[130]
Echinacoside	Bone marrow cells (BM) and bone marrow stromal cells (BMSCs)	Stimulate BMSC proliferation and produce GM-CSF, thereby stimulating and promoting the proliferation and differentiation of HSC, activate the PI3K pathway in 5-FU-inhibited bone marrow cells.	[131]
3,3′-dimethyl ellagic acid-4′-*O*-glucoside (DMAG)	HEL and Meg-01 megakaryocyte cell	By directly binding to core targets such as ITGA2B, ITGB3, TLR2, etc., key signaling pathways such as PI3K Akt are activated to promote megakaryocyte differentiation and platelet production.	[66,68,132]

**Table 3:** Study on the activity of glycoside compounds in the treatment of thrombocytopenia.

Glycosides	Test Model	Mechanism	Reference
Panax diol saponins	Cyclophosphamide-induced bone marrow suppression Kunming mouse model	upregulates *C-kit* and *GATA-1* transcription factors in bone marrow cells by phosphorylating intracellular MAPK signaling pathways of MEK and ERK and upregulating C-kit and GATA-1 transcription factors in bone marrow cells.	[167,168]
	BALB/c mouse model of immune thrombocytopenia (ITP) was established with an anti-platelet antibody.	Inhibit the phagocytosis of exogenous red blood cells by peritoneal macrophages in ITP mice, and inhibit the phagocytosis of antibody-coated platelets by regulating immunity.	
Ginsenosides	Cyclophosphamide-induced bone marrow suppression in Balb/c mice	Promote more cells to enter the normal cell cycle through the G1 phase checkpoint, regulate the balance of *bcl-2/bax*, inhibiting the expression of caspase-3, thereby inhibiting the apoptosis of BMNC.	[91,120]
Panax notoginsenosides	Bone marrow suppression in mice induced by 60Co γ-ray irradiation	The hemoglobin, the total number of white blood cells, and the colony yield of CFU-E and CFU-GM in the peripheral blood of irradiated mice were increased.	[168]
Icariin	ITP mouse model induced by injection of guinea pig anti-mouse platelet serum Radiation-injured mice (irradiated with 60 Co) Fanca +/+, Fanca −/−, Fancd2 +/+ and Fancd2 −/− mice	Promote megakaryocytes to produce platelets; promote hematopoiesis. Promote the formation of CFU-GM colonies in bone marrow and spleen. By stimulating the activity of SIRT6, the function of HSC was improved, and the ability of mutant stem cells to form colony-forming units (CFU) in vitro was enhanced.	[14,169]
Quercetin-3-glucuronide (QG)	Immune-mediated bone marrow failure mouse model	Up-regulate the expression of the PI3K/AKT pathway, reduce platelet apoptosis in mice with immune bone marrow failure, and participate in the regulation of partial platelet activation function.	[170]
Rhodiola glycoside	Cyclophosphamide (CTX) -induced bone marrow injury in mice	Increase the level of hematopoietic factors, promote bone marrow hematopoiesis, and then increase white blood cells and platelets in peripheral blood.	[19]
Paeoniflorin	Mouse model of blood deficiency syndrome was induced by irradiation.	Promote the secretion of hematopoietic factors (G-CSF, GM-CSF, PDGF-α) by bone marrow stromal cells and inhibit the secretion of hematopoietic inhibitory factor (M-CIP).	[10]
Rehmannioside D	Blood deficiency in mice induced by thyroxine, reserpine, and cyclophosphamide	Increase the number of white blood cells, platelets, reticular cells, and bone marrow DNA content in blood-deficient mice.	[56,58]
Astragaloside IV	Cyclophosphamide-induced bone marrow suppression/immunosuppression mouse model	Activation of the HIF-1α/NF-κB signaling pathway enhances macrophage and lymphocyte function.	[171]

## Data Availability

No new data were created or analyzed in this study. Data sharing is not applicable to this article.

## Abbreviations

The following abbreviations are used in this manuscript:

ACS	Acute coronary syndrome
AKT	Protein kinase B
ALT	Alanine Aminotransferase
AMPK	AMP-activated protein kinase
ARE	Antioxidant response element
BUN	Blood urea nitrogen
CSF	Colony-stimulating factor
DHC	Dihydrocapsaicin
EPO	Erythropoietin
EQOL	Enhanced quality of life
ERK	Extracellular signal-regulated kinase
FOS	Fos proto-oncogene
GATA	GATA binding protein
GH	Growth hormone
GR	Glucocorticoid receptor
HIF	Hypoxia-inducible factor
DMAG	3,3′-Dimethyl Ellagic Acid-4′-*O*-glucoside
HO-1	Heme oxygenase-1
HSPC	Hematopoietic stem and progenitor cell
IFN	Interferon
IL	Interleukin
ITP	Immune thrombocytopenia
JAK	Janus kinase
MAPK	Mitogen-activated protein kinase
MDS	Myelodysplastic syndrome
MEK	MAPK/ERK kinase
NF-κB	Nuclear factor kappa-B
PDGF	Platelet-derived growth factor
PI3K	Phosphatidylinositol 3-kinase
PK	Pharmacokinetics
PNSs	Panax notoginseng saponins
PR	Progesterone receptor
JAK2/STAT3	Janus Kinase 2/Signal Transducer and Activator of Transcription 3
KCR	Kaempferol-3-*O*-α-L-(4″-*E*-*p*-coumaroyl) rhamnoside
MEK/ERK	MAPK/ERK kinase/Extracellular signal-Regulated Kinase
RANKL	Receptor activator of nuclear factor-κB ligand
ROS	Reactive oxygen species
PDS-C	Panaxadiol Saponin Component
PI3K/AKT	Phosphatidylinositol 3-kinase/Protein kinase B
PLT	Platelet
SAR	Structure–activity relationship
TCM	Traditional Chinese Medicine
